# Awareness, knowledge and screening for cervical cancer among women of a faith-based organization in Nigeria

**DOI:** 10.11604/pamj.2021.39.200.23761

**Published:** 2021-07-14

**Authors:** Lawreta Ijeoma Abugu, Evelyn Nwanebe Nwagu

**Affiliations:** 1 University of Nigeria Nsukka, Department of Human Kinetics and Health Education, Nsukka, Nigeria

**Keywords:** Awareness, knowledge, screening, cervical cancer, women

## Abstract

**Introduction:**

cervical cancer is the second most frequent cancer among women in Nigeria. With proper screening, the disease can be detected early enough and properly managed. However, there seems to be limited knowledge of cervical cancer among women and several barriers might prevent them from accessing the screening services. We determined the awareness, knowledge and screening for cervical cancer among women of a faith-based organization in Nigeria.

**Methods:**

we employed descriptive survey research design and purposively sampled a Catholic Parish in Nsukka Local Government Area of Enugu State, Nigeria. All consenting women in the Parish were used for the study. We utilized researchers' structured instrument titled ‘Awareness and knowledge about Cervical Cancer and Screening’ to collect data. Frequencies, percentages and logistic regression were employed for data analysis.

**Results:**

awareness of cervical cancer (70.8%) and its screening (68.1%) are high but there is generally poor level of knowledge (65.3%) of different aspects of cervical cancer among the women. Age (aOR: 7.183, 95% CI 1.769-29.168; p=.006), number of children (aOR: .074, 95% CI .009- .631; p=.017), and occupation (aOR: .032, 95% CI .004-.260; p= .001 and aOR: .050, 95% CI .007-.347; p=.002) were significantly associated with knowledge of cervical cancer. Majority of the women studied had never been screened for cervical cancer (91.7%) and the reasons for not screening ranged from lack of knowledge of; where to go for screening (69.7%) and the importance of being screened (40.9%) to not feeling susceptible to cervical cancer (18.2%).

**Conclusion:**

there was generally poor knowledge of cervical cancer and its screening; and very poor screening practice. There is, therefore, an urgent need to improve women´s knowledge of cervical cancer and address the identified barriers in order to improve screening practices of the women.

## Introduction

Cervical cancer is the most common gynaecological cancer among women in sub-Saharan Africa [1]. In Nigeria, cervical cancer is the second most frequent cancer among women after breast cancer. The current prevalence of cervical cancer in Nigeria is 250/100,000 population. Nigeria recorded 102,079 cases of cancer, out of which 14,089(13.8%) is for cervical cancer. Mortality showed that cervical cancer related deaths was 8,240 (11.5%) of all cancers in Nigeria [2]. With proper screening, the disease can be detected early enough and properly managed. Several screening methods for cervical cancer are available which includes Pap smear, and visual inspection with acetic acid (VIA). However, VIA as reported by Pandigini and colleagues is the only currently available, affordable and sustainable large scale method for cervical cancer screening [3]. However, most women might not be aware of this health service or might have several barriers preventing them from adopting cervical cancer screening services.

Studies have shown that there is limited knowledge on this important health preventive and promotive service [3-5]. There is therefore an urgent need for information and education on awareness of cervical cancer and early detection measures [6]. Also women face several barriers to the uptake of the services which ranges from personal, financial, health system to cultural barriers [1, 6, 7]. This might also be the case with women in Nsukka Local Government Area. People from Nsukka belong to the Igbo ethnic group in Nigeria. In Nsukka however, the practice of health screening is usually poor. Many cultural beliefs might hinder women from partaking in screening programmes for cervical cancer. People do not visit health facilities until at advanced stage of their illness. Herbal medicines and use of the patent medicine dealers´ services are the most prevalent form of health service readily used by indigenes. These could result to many chronic and preventable diseases including cervical cancer.

Involving faith based organization in prevention programmes like cervical cancer prevention is an effective means of achieving good health among community members. This is because any programme that is church based has a lot of impact on the masses [8]. Religious leaders are among the most powerful stakeholders in the society so any prevention strategy using the church will go a long way in achieving its purpose. This study therefore explored the awareness, knowledge and screening for cervical cancer as well as barriers to uptake of screening services among women.

## Methods

**Study design and setting:** we adopted a descriptive survey research design and carried out the study in Nsukka town, a semi-urban community and the headquartes of Nsukka Local Government Area of Enugu state, Nigeria. The town is about 50 kilometers from Enugu, the state capital. The inhabitants are predominantly Igbos, mostly Christians and traders, with an appreciable number of civil servants because of a federal university in the town, and a few farmers. Among the Christian denominations in the community, the Roman Catholic Church is the most predominant. Catholic Parishes in the area usually have women group known as Catholic Women Organisation. This group celebrates Mother´s day annually during which several programmes including health programmes use to be part of the activities to mark the celebration. We conducted the study in March, 2019 during the activities to mark the 2019 Mother´s day celebration of the Roman Catholic Church. Free medical screening was part of the activities for the day´s celebration in St Mary´s Parish, Amogbo, Nsukka. Cervical cancer awareness and screening was part of the programme for the Mother´s day celebration.

**Study population:** population for the study consisted of all the 201 women aged 20 years and above who presented during the medical screening exercise to mark the year´s mother´s day celebration at St Mary´s Parish, Amogbo, Nsukka. St Mary´s Parish Amogbo was purposively chosen for the study because it is a big parish at the heart of the town with people of different categories of interest residing and attending church services at the Parish. All eligible participants who consented to the study were recruited and used, therefore no sampling was done.

**Data collection:** researchers structured instrument titled ‘Awareness and Knowledge of Cervical Cancer Screening’ was used to collect data. The instrument was designed based on findings from literature search on what is known about cervical cancer and its screening procedures. The instrument consists of two sections; section A was used to elicit information on the participants´ demographic characteristics, while section B elicited information on participants awareness, knowledge and screening practices for cervical cancer.

**Statistical analysis:** data were analysed using SPSS version 21. One hundred and forty four (144) correctly filled out questionnaires were used for analysis. Only the participants who claimed to be aware of cervical cancer were asked to respond to further question on their source of information, knowledge of the disease condition, risk factors, symptoms and screening for cervical cancer (see Table 1). For factors associated with knowledge of cervical cancer, we performed both univariable and multivariable regression analysis. Only variables with a p value less than .05 in the univariable analysis were included in the multivariable model.

**Table 1:** awareness and knowledge of cervical cancer

Variable	Frequency (f)	Percentage (%)
**Ever heard of cervical cancer (n=144)**		
Yes	102	70.8
No	42	29.2
**Source of information (n-102)**		
Health worker	66	64.7
Media	36	35.3
Family	18	17.6
Literature	10	9.8
**Knowledge of concept of ca cervix (n=102)**		
A very dangerous cancer affecting women in their reproductive age	78	76.5
A type of cancer that affects all women irrespective of age	44	43.1
Cancer of cervix that can lead to death of women	84	82.4
Cancer that is common in both men and women	6	5.9
A type of cancer that can be treated if detected early	88	86.3
**Knowledge of causes/risk factors (n=102)**		
Post menopause	22	21.6
Having many sexual partners	60	58.6
Increase in age above 45 years	38	37.3
Having sex at early age	48	47.1
Giving birth to many children	44	43.1
History of sexual transmitted diseases	50	49.0
Poor menstrual hygiene	44	43.1
Lack of cervical cancer screening	52	51.0
**Knowledge of signs and symptoms (n=102)**		
Pain in the pelvic region	68	66.6
Bleeding after intercourse	70	68.6
Abnormal vaginal discharge	48	47.1
Post-menopausal bleeding	40	39.2
Abdominal discomfort	42	41.2
Spotting blood in between menstruation	38	37.3
Heavy menstrual bleeding	38	37.3
**Ever heard of screening for cervical cancer**		
Yes	98	68.1
No	46	31.9
**Knowledge of screening for cervical cancer (n=98)**		
Test to find out whether you have cervical cancer	80	81.6
Test to find out whether you will likely develop cervical cancer in future	76	77.6
Test to find out whether you have abnormal cervix	18	18.4
Test to determine your sexual activity	4	4.1
**Level of knowledge**		
Poor	94	65.3
Good	50	34.7

**Ethical considerations:** we explained the purpose of the research to the women after which their consent to participate in the study was sought. Participants were informed that participation is strictly voluntary and that they are free to withdraw from the study any time they so wish. Only consenting women were used. We obtained consent both verbally and in writing. Ethical approval for the study was granted by the research ethics committee, Faculty of Education University of Nigeria, Nsukka.

## Results

**Socio demographic characteristics of participants:**Table 2 shows that most of the respondents earn less than 60,000 naira as monthly income (79.2%) were married (76.4%) aged 41-60 years (65.3%) and have four and above children (63.9%). The table also show that greater percentage of the respondents were civil servants (47.9%) and have tertiary education (47.2%).

**Table 2:** socio-demographic characteristics of respondents (n=144)

Characteristic	Frequency (f)	Percentage (%)
**Age range (years)**		
20-40	42	29.2
41-60	94	65.3
Above 60	8	5.6
**Highest level of education**		
Primary	36	25.0
Secondary	40	27.8
Tertiary	68	47.2
**Marital status**		
Married	110	76.4
Widowed	28	19.4
Single	6	4.2
**Number of children**		
None	14	9.7
1-3	38	26.4
4 and above	92	63.9
**Occupation**		
Civil servant	69	47.9
Self employed	60	41.7
Unemployed	15	10.4
**Monthly income (Naira)**		
Less than 60,000	114	79.2
60,000 and above	30	20.8

**Awareness and knowledge about cervical cancer and its screening:** seventy point eight per cent of the women have ever heard of cervical cancer and health workers (64.7%) were majorly the source of their information (Table 1). Furthermore, on the knowledge of concept of cervical cancer, 43.1 per cent wrongly said that it affects all women irrespective of age and 5.9 percent were wrongly of the view that it affects both men and women. Less than half of the respondents knew the risk factors of cervical cancer, except the risk of many sexual partners (58.6%) and lack of screening (51.0%) which slightly above half of the participants knew of. Majority of the respondents knew that bleeding after intercourse (68.6%) and pain in the pelvic region (66.6%) were the signs and symptoms of cervical cancer. The other symptoms were known by less than half of the respondents. Again 68.1 percent of respondents had heard of screening for cervical cancer and majority of them (77.6%) identified that cervical cancer screening is a test to find out whether you will likely develop cervical cancer in future. Greater proportion of respondents (65.3%) had poor level of knowledge of cervical cancer.

**Factors associated with knowledge of cervical cancer:**Table 3 shows univariable and multivariable analysis of factors associated with knowledge of cervical cancer. In the multivariable analysis, women aged 41-60 years were 7 times more likely to have knowledge of concept of cervical cancer than those aged 20-40 years (aOR: 7.183, 95% CI 1.769-29.168; p=.006). Those with four children and above were 92.6% less likely to have concept knowledge than those with no child (aOR: .074, 95% CI .009- .631; p=.017). Self employed and unemployed women were 96.8% and 95% respectively less likely to have knowledge of concept of cervical cancer than civil servants (aOR: .032, 95% CI .004-.260; p= .001 and aOR: .050, 95% CI .007-.347; p=.002). On knowledge of causes of cervical cancer, women who were self-employed were 80.3% less likely to have knowledge than civil servants (aOR: .197, 95% CI .055-.707; p=.013). For knowledge of signs and symptoms, women with 1-3 children and 4 children and above were 75.7% and 80.4% less likely to have knowledge of signs and symptoms of cervical cancer than those with no child (aOR: .243, 95% CI .060 - .989; p=.048 and aOR: .196, 95% CI .051 - .748; p=.017). For knowledge of screening for cervical cancer, women who were self employed and unemployed were 98.9% and 95.2% respectively less likely to have knowledge of cervical cancer screening (aOR: .011, 95% CI .001-.091; p=.000 and aOR: .048, 95% CI .008-.300; p=.001) respectively.

**Table 3:** univariable and multivariable analysis of factors associated with knowledge of cervical cancer

Factors	Univariable		Multivariable	
KNOWLEDGE OF CONCEPT	OR(95%CI)	P- value	aOR(95% CI)	P- value
**Age category**		.135		
20-40 yearsa				
41-60 years	2.188(1.016-4.711)	.045	7.183(1.769-29.168)	.006*
Above 60 years	1211606149(.000)	.999		
**Level of education**		.000		
Primary Educationb				
Secondary Education	.978(.395-2.419)	.961	1.485(.387-5.701)	.565
Tertiary Education	6.000(2.234-16.117)	.000	.770(.101-5.870)	.801
**Number of children**		.029		
0c				
1-3	.889(.157-5.026)	.894	.623(.079-4.879)	.652
4 and above	.284(.060-1.347)	.113	.074(.009-.631)	.017*
**Occupation**		.000		
Civil servantd				
Self employed	.054(.017-.167)	.000	.032(.004-.260)	.001*
Unemployed	.092(.022-.391)	.001	.050(.007-.347)	.002*
**Monthly Income**				
Less than N 60,000e				
N 60,000 and above	3.250(1.058-9.985)	.040	.753(.113-5.026)	.770
**KNOWLEDGE OF CAUSES**				
**Level of education**		.002		
Primary Educationb				
Secondary Education	1.250(.388-4.027)	.709	1.085(.317-3.710)	.897
Tertiary Education	4.444(1.639-12.051)	.003	1.360(.351-5.273)	.657
**Occupation**		.000		
Civil servantd				
Self employed	.158(.066-.382)	.000	.197(.055-.707)	.013*
Unemployed	.374(.109-1.291)	.120	.410(.111-1.513)	.181
**KNOWLEDGE OF SIGNS AND SYMPTOMS**				
**Level of Education**		.009		
Primary Educationb				
Secondary Education	2.143(.708-6.483)	.177	1.650(.506-5.379)	.406
Tertiary Education	4.444(1.639-12.051)	.003	1.446(.338-6.182)	.619
**Number of children**		.014		
0c				
1-3	.233(.061-.886)	.032	.243(.060-.989)	.048*
4 and above	.158(.045-.547)	.004	.196(.051-.748)	.017*
**Occupation**		.002		
Civil servantd				
Self employed	.257(.117-.567)	.001	.318(.090-1.115)	.073
Unemployed	.374(.109-1.291)	.120	.296(.072-1.212)	.090
**KNOWLEDGE OF SCREENING**				
**Level of education**		.000		
Primary Educationb				
Secondary Education	1.023(.413-2.530)	.961	.374(.107-1.304)	.123
Tertiary Education	9.395(3.490-25.183)	.000	.248(.032-1.909)	.180
**Number of children**		.016		
0c				
1-3	.625(.196-3.380)	.585	1.179(.156-8.921)	.873
4 and above	.217(.046-1.024)	.054	.212(.030-1.490)	.119
**Occupation**		.000		
Civil servantd				
Self employed	.031(.010-.097)	.000	.011(.001-.091)	.000*
Unemployed	.092(.022-.391)	.001	.048(.008-.300)	.001*
**Monthly Income**				
Less than N 60,000e				
N 60,000 and above	4.397(1.439-13.439)	.009	.680(.119-3.873)	.664

Ref Groups: Age category=20-40 yearsa ; Level of Education= Primary Educationb ; Number of children= 0c ; Occupation= Civil servantd ;Monthly income= Less than N 60,000e CI = Confidence Interval; aOR= adjusted Odds Ratio

**Barriers to screening for cervical cancer:** most of the respondents (91.7%) had never been screened for cervical cancer (Table 4). The most reported barrier to screening was lack of knowledge of where to go for screening (69.7%), lack of knowledge of screening importance (40.9%) and lack of money (36.4%). Also, 19.7 per cent were afraid of what the result could be while 18.2 per cent did not think they can ever develop cervical cancer.

**Table 4:** barriers to screening among women

Variable	Frequency (f)	Percentage (%)
**Ever screened for ca (n=144)**		
Yes	12	8.3
No	132	91.7
**Barriers to screening (n=132)**	0	
Lack of knowledge of where to go for screening	92	69.7
Lack of money	48	36.4
Afraid of what the result could be	26	19.7
Exposing private part to health worker will embarrass me	2	1.5
I do not have time to go for the screening	12	9.1
It is not necessary for me to have cervical cancer screening	4	3.0
The health care workers in the clinic are unfriendly to patients	0	0
Lack of knowledge of the importance of screening	54	40.9
I do not think I can ever develop cervical cancer	24	18.2

## Discussion

Most of our participants were aware of cervical cancer, but many lacked proper knowledge of the concept, causes and signs and symptoms of the disease as shown in Table 1. Majority of the participants (77.6%) also know that screening for cervical cancer is a test to find out whether you will likely develop the disease in future. In other words, awareness and knowledge of screening for cervical cancer is relatively high among participants. However, being aware of the existence of a disease condition is good but awareness without proper knowledge of features and factors associated with a disease condition will not produce any positive result in terms of prevention of such disease.

Similar studies had reported low awareness and knowledge of cervical cancer. For instance, Ndikom and Ofi reported low knowledge of cervical cancer among women in Ibadan, Nigeria [9]. Low awareness and poor knowledge of cervical cancer among rural Nigerian women was also reported by Abiodun, Fatungase and Olu-Abiodun [10]. In Mangalore City, majority of the women (81.9%) had poor knowledge of cervical cancer [11]. In the current study, though awareness is high, the knowledge of the several aspects of cervical cancer is low. The high awareness shown in this study could be attributed to the fact that greater percentage of the study participants had tertiary education so may have heard of cervical cancer without really knowing the details of the disease.

Awareness and knowledge of screening for cervical cancer is relatively high in this study. Many similar studies had reported low awareness and poor knowledge regarding screening for cervical cancer [10, 11]. However, in a study of Female Medical Students in the Commonwealth of Dominica, most students were aware of cervical cancer screening [3]. This exceptional finding could be because the respondents were medical students and had as such become very familiar with screening services for cancer of the cervix in the course of their training.

Our study found that age, number of children and occupation were significantly associated with knowledge of different aspects of cervical cancer. Women aged 41-60 years were 7 times more likely to have knowledge of concept of cervical cancer than those aged 20-40 years (aOR: 7.183, 95% CI 1.769-29.168; p=.006). This could be because increasing age increases one´s life experiences including knowledge of cervical cancer. Age was also significantly associated with awareness of cervical cancer in north central Nigeria [12] where women aged 55 years and older tend to have more awareness of cervical cancer. However, this study found out that those with four children and above were 92.6% less likely to have knowledge of concept of cervical cancer than those with no child (aOR: .074, 95% CI .009- .631; p=.017). This finding is unexpected and contradicts some other recent finding. For instance, Hycinth *et al*. reported significant association of number of children and awareness of cervical cancer in Nigeria [12] where those women with four to six children were more likely to have awareness than those with lesser number of children. It may be that as the women´s parity increases, they become encumbered with many responsibilities which could lead to their forgetting what they use to know.

A study by Glynnn reported that multiparity was associated with poorer memory function [13]. Self employed and unemployed women were 96.8% and 95% respectively less likely to have knowledge of concept of cervical cancer than civil servants (aOR: .032, 95% CI .004-.260; p= .001 and aOR: .050, 95% CI .007-.347; p=.002). The finding could be because civil servants may be more exposed to health issues and may be associating with health care workers more hence they are more likely to have cervical cancer knowledge. Being a civil servant was shown to be significantly associated with cervical cancer screening in a similar study in north east Nigeria [14]. Most of the women in our study (91.7%) had never been screened for cervical cancer (Table 4). This is an unfortunate finding which can predispose the women to the disease condition when it could otherwise have been prevented. This finding is however consistent with several other studies showing poor level of screening for cervical cancer [3, 10, 11, 15].

The most reported barrier to screening among our participants was lack of knowledge of where to go for screening (69.7%), lack of knowledge of screening importance (40.9%) and lack of money (36.4%). Also, 19.7 per cent were afraid of what the result could be while 18.2 per cent did not think they can ever develop cervical cancer. Similar barriers were reported in other studies. For instance, Ndikom and Ofi report barriers such as unavailability of screening services in most facilities, ignorance, and illiteracy, belief in not being at risk, financial constraint and fear of positive result among rural Nigerian women [9]. Also hospital related, geographical, economic, educational and psychosocial barriers were reported among rural Nigerian women in South West, Nigeria by Onyenwenyi and Mchunu [16].

Our findings seem to point to the need for individualized and targeted health education for women in the area of study. Women should be given relevant and consistent information about cancer of the cervix individually and in small groups. Mass media campaign and general prevention information is not enough to address prevention of cancer of the cervix [17]. Health care workers should make good use of any opportunity available to them to teach and screen women especially older women for cervical cancer [18], informing them where they will get such screening services in order to reduce the high incident of cancer of the cervix. In order to address some of the barriers to screening for cervical cancer as identified in this study, stakeholders like traditional and religious leaders could be used to advocate for more uptake of screening services by women in the community. Their involvement could go a long way in improving cervical cancer screening uptake. Community wide education on cervical cancer and its prevention is also a good way to improve knowledge and screening for cervical cancer as most men would be involved when it becomes a community project.

This study has been able to provide literature on what women of a faith based organization in Nigeria know of cervical cancer and its screening. The study equally identified factors associated with poor knowledge of cervical cancer among the study population. However, the study relied only on quantitative data which has no means of verification of responses. This becomes a limitation of the study. Incorporating both quantitative and qualitative data could have provided a more robust explanations of the knowledge of cervical cancer as well as barriers to screening among the study group. Therefore future research needs include exploration of the subject matter by means of a mixed method approach.

## Conclusion

Our findings showed that even though awareness of cervical cancer and its screening is high, there is generally low level of knowledge of different aspects of cervical cancer among women of a faith based organization in Nigeria. Age, number of children and occupation were significantly associated with knowledge of different aspects of cervical cancer. Majority of the women studied had never been screened for cervical cancer and the reasons for not screening ranged from lack of knowledge of where to go for screening to not feeling susceptible to cervical cancer. There is therefore urgent need to improve women´s knowledge of cervical cancer and address the identified barriers in order to improve screening practices of the women. We therefore recommend that health educators and health care workers should utilize every opportunity with women to educate them on taking preventive health actions like screening services for prevention of cervical cancer.

### What is known about this topic

- Cervical cancer is the second most common cause of death due to cancer among women in Nigeria;
- Cervical cancer can be prevented by early diagnosis through regular screening for changes in the cervix suggestive of cervical cancer.

### What this study adds

- Women are aware of cervical cancer but lack knowledge of different aspects of cervical cancer;
- There is generally poor screening practices for cervical cancer among women in the study area;
- Lack of knowledge on the importance of screening and where to access screening services made women not to screen for cervical cancer.
